# MCP-3 as a prognostic biomarker for severe fever with thrombocytopenia syndrome: a longitudinal cytokine profile study

**DOI:** 10.3389/fimmu.2024.1379114

**Published:** 2024-05-15

**Authors:** Zishuai Liu, Chenxi Zhao, Hong Yu, Rongling Zhang, Xiaoyu Xue, Zhouling Jiang, Ziruo Ge, Yanli Xu, Wei Zhang, Ling Lin, Zhihai Chen

**Affiliations:** ^1^ National Key Laboratory of Intelligent Tracking and Forecasting for Infectious Diseases, Beijing Ditan Hospital, Capital Medical University, Beijing, China; ^2^ Department of Infectious Diseases, Yantai Qishan Hospital, Yantai, Shandong, China

**Keywords:** severe fever with thrombocytopenia syndrome, longitudinal cytokine profile, MCP-3, biomarker, predictive

## Abstract

**Introduction:**

Severe fever with thrombocytopenia syndrome (SFTS) is characterized by a high mortality rate and is associated with immune dysregulation. Cytokine storms may play an important role in adverse disease regression, this study aimed to assess the validity of MCP-3 in predicting adverse outcomes in SFTS patients and to investigate the longitudinal cytokine profile in SFTS patients.

**Methods:**

The prospective study was conducted at Yantai Qishan Hospital from May to November 2022. We collected clinical data and serial blood samples during hospitalization, patients with SFTS were divided into survival and non-survival groups based on the clinical prognosis.

**Results:**

The levels of serum 48 cytokines were measured using Luminex assays. Compared to healthy controls, SFTS patients exhibited higher levels of most cytokines. The non-survival group had significantly higher levels of 32 cytokines compared to the survival group. Among these cytokines, MCP-3 was ranked as the most significant variable by the random forest (RF) model in predicting the poor prognosis of SFTS patients. Additionally, we validated the predictive effects of MCP-3 through receiver operating characteristic (ROC) curve analysis with an AUC of 0.882 (95% CI, 0.787-0.978, *P <*0.001), and the clinical applicability of MCP-3 was assessed favorably based on decision curve analysis (DCA). The Spearman correlation analysis indicated that the level of MCP-3 was positively correlated with ALT, AST, LDH, α-HBDH, APTT, D-dimer, and viral load (*P*<0.01).

**Discussion:**

For the first time, our study identified and validated that MCP-3 could serve as a meaningful biomarker for predicting the fatal outcome of SFTS patients. The longitudinal cytokine profile analyzed that abnormally increased cytokines were associated with the poor prognosis of SFTS patients. Our study provides new insights into exploring the pathogenesis of cytokines with organ damage and leading to adverse effects.

## Graphical Abstract

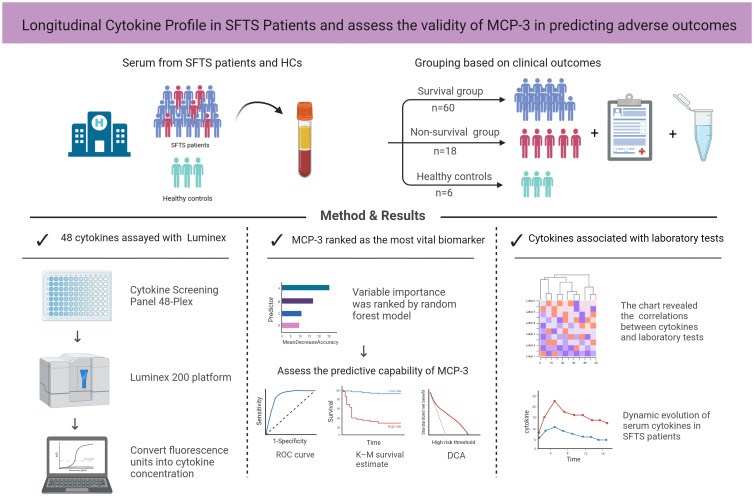

## Introduction

Severe fever with thrombocytopenia syndrome (SFTS) is an acute tick-borne disease resulting from SFTS virus (SFTSV) infection (1). Reports indicate that SFTSV can also be transmitted among family members through close contact (2), involving exposure to blood and aerosols. Some patients were infected through close contact with sick domestic cats infected with SFTSV (3, 4). Since its first report in 2009, SFTSV has been spreading across several countries and regions, including Japan, South Korea, Taiwan, Vietnam, and Thailand (5–9). As of 2019, 13824 laboratory-confirmed cases have been reported across 25 provinces in China (10). Despite a decrease from 30% in the early stages due to improved understanding, the case fatality rate remains high at 6.1%-21.8% (11, 12), several studies showed the efficiency of favipiravir in treating SFTS patients (13, 14). Timely recognition and assessment of patients with severe conditions, providing sufficient and comprehensive supportive measures, are crucial for improving outcomes in individuals with SFTS. Therefore, accurately identifying critically ill patients at an early stage is of utmost importance in clinical practice.

Currently, the mechanism underlying the severity of SFTS is not well understood. Nevertheless, multiple studies have suggested a close association between the poor prognosis of SFTS patients and cytokine storm (15, 16). Numerous studies have explored the risk factors associated with adverse outcomes in SFTS patients, including clinical symptoms, signs, and laboratory parameters (17). However, only a limited number have delved into the role of cytokines in predicting a poor prognosis.

Monocyte chemoattractant protein-3 (MCP-3) is a chemokine encoded by the C-C motif chemokine ligand 7 (CCL7) gene (18). It is mainly expressed in monocytes, fibroblasts, and T cells. MCP-3 functions by attracting monocytes and eosinophils, but not neutrophils (19). Additionally, it enhances the anti-tumor activity of monocytes and binds to CC motif chemokine receptor (CCR) 1, CCR2, and CCR3, thereby influencing immune cell function (20). Previous studies have shown the robust performance of MCP-3 in identifying disease severity and predicting poor prognosis in various infectious and non-infectious diseases. For example, Sun L et al. successfully differentiated between mild and severe asthma patients using MCP-3 (21). Findings by Yang indicated a significant correlation between high serum MCP-3 concentrations and the severity of COVID-19 patients (22). Although the role of MCP-3 in predicting an unfavorable prognosis for SFTS patients remains unexplored, previous research on SFTS has indicated the crucial significance of MCP-3 in prognosticating a fatal outcome in SFTS patients. For instance, studies suggest that the MCP-3 binding protein CCR2 is the receptor for SFTSV to enter the host (23). Other studies have demonstrated that monocytes, one of the primary target cells of SFTSV, are closely associated with severe disease in patients (24). Additionally, eosinophils, one of the central chemotactic cells of MCP-3, have been identified as an independent risk factor for adverse outcomes in patients with SFTS (25). Similarly, research has shown increased intrathecal expression of CCL7 in patients with tick-borne encephalitis (26).

It is reasonable to suspect that MCP-3 plays a significant role in the cytokine storm triggered by SFTSV infection. Furthermore, the concentration of MCP-3 closely correlates with the severity of SFTS disease, serving as a reliable indicator for predicting the prognosis of SFTS patients. To validate this hypothesis, we conducted a study using the Luminex technique to assess 48 cytokines in 78 SFTS patients with varying clinical outcomes. We also monitored the dynamic changes in these 48 factors as the disease progressed. Our study provides initial evidence of the valuable predictive and prognostic role of MCP-3 in SFTS disease.

## Materials and methods

### Study design and patients

**Figure 1:**
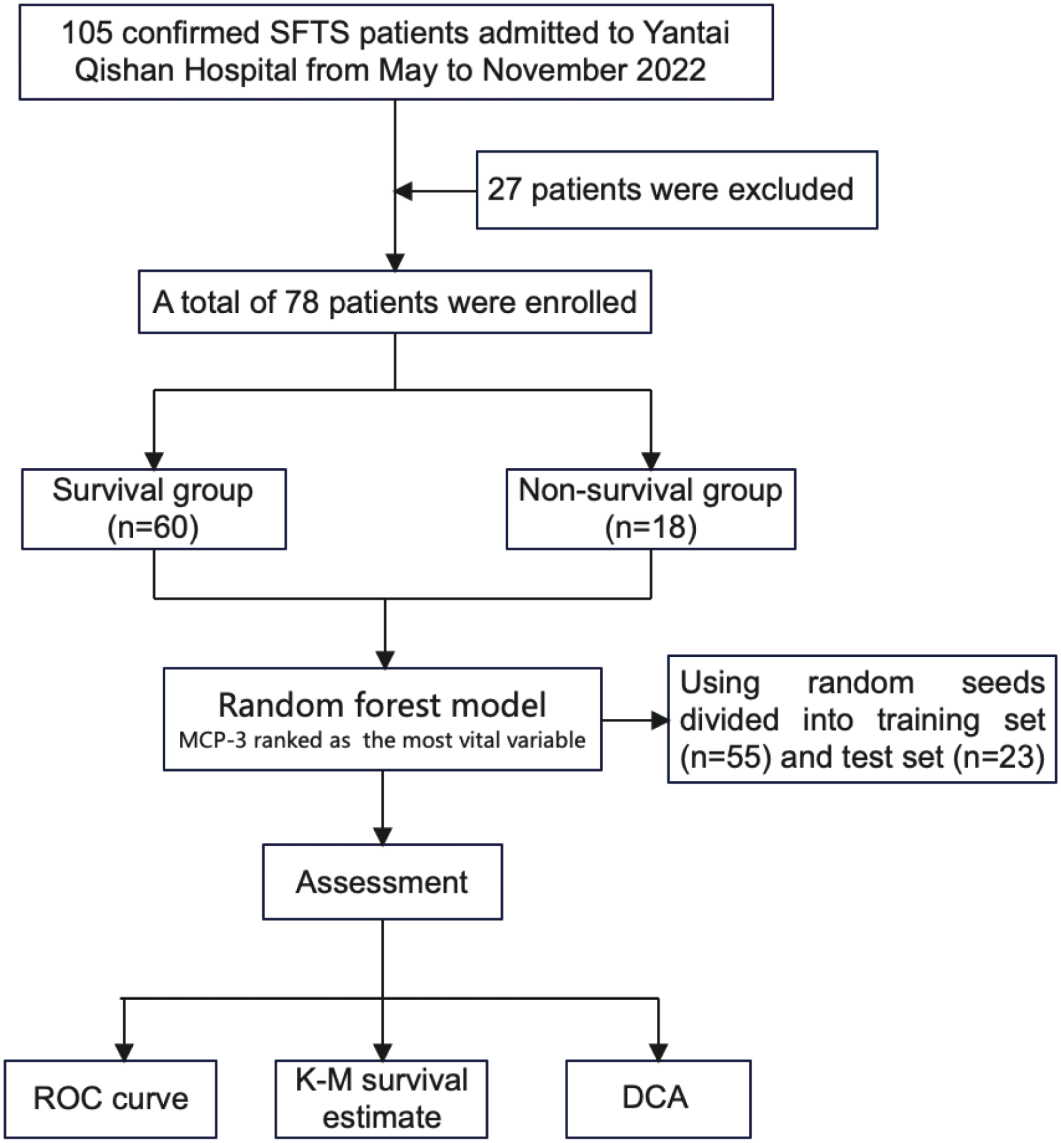
Workflow diagram of study design.

This prospective study was performed from May to November 2022 at Yantai Qishan Hospital and included patients diagnosed with SFTS who were admitted to the hospital during this period and met the inclusion and exclusion criteria. The diagnostic criteria for SFTS were positive real-time fluorescent polymerase chain reaction (RT-PCR) for SFTS virus RNA during hospitalization. The exclusion criteria were: (1) Patients with other viral infections; (2) Previous leukemia, idiopathic thrombocytopenic purpura, and other blood system diseases; (3) Previous autoimmune diseases. In total, 78 patients meeting the enrollment criteria were included in the final analysis, of whom 18 had a fatal outcome. The endpoints observed in this study were defined as discharge or death (the complete study schematic chart is shown in **Figure 1**). The Ethics Committee of the lead center, Beijing Ditan Hospital, Capital Medical University, approved this study, which strictly adhered to the principles of the Declaration of Helsinki. Patients were informed and signed the informed consent.

### Data collection

Patient information, encompassing baseline demographics, clinical characteristics, and laboratory parameters, was extracted from medical records upon admission. Acute phase means within 7 days post-symptom onset (24, 27). We acquired serial peripheral blood samples from 78 SFTS patients 12 hours after admission and every other day during their hospitalization. These serum samples were promptly centrifuged and stored in a freezer at -80°C until further analysis. Besides, six volunteers were selected as healthy controls and matched for gender and age (**Supplementary Table 1**).

### Cytokine assay

The subjects’ serum levels of 48 cytokines were quantified with the Bio-Plex Pro Human Cytokine Screening Panel 48-Plex (Bio-Rad, Hercules, California, USA; 12007283) following the manufacturer protocol. Plate analysis was conducted using the Luminex 200 platform (Milliplex Analyst, version 5.1). We applied a standard curve with known recombinant human concentrations of cytokine to convert fluorescence units to cytokine concentration units (pg/mL). The assay examined the following cytokines: interleukin (IL)-1α, IL-1β, IL-1ra, IL-2, IL-2Rα, IL-3, IL-4, IL-5, IL-6, IL-7, IL-9, IL-10, IL-12(p40), IL-12 (p70), IL-13, IL-15, IL-16, IL-17, IL-18, leukemia inhibitory factor (LIF), granulocyte colony-stimulating factor (G-CSF), granulocyte macrophage colony-stimulating factor (GM-CSF), macrophage colony-stimulating factor (M-CSF), stem cell factor (SCF), stem cell growth factor (SCGF)-β, interferon (IFN)-γ, IFN-α2, tumor necrosis factor (TNF)-α, TNF-β, tumor necrosis factor-related apoptosis-inducing ligand (TRAIL), basic fibroblast growth factors (FGF), vascular endothelial growth factor (VEGF), platelet derived growth factor-BB (PDGF-BB), hepatocyte growth factor (HGF), β-nerve growth factor (NGF), CXCL1 (GRO-α), CXCL8 (IL-8), CXCL9 (MIG), CXCL10 (IP-10), CXCL12 (SDF-1α), CCL2 (MCP-1), CCL3 (MIP-1α), CCL4 (MIP-1β), CCL5 (RANTES), CCL7(MCP-3), CCL11 (Eotaxin), CCL27 (CTACK), macrophage migration Inhibitory Factor (MIF). In statistical analyses, cytokines were excluded if over 50% of values fell outside the upper or lower limits of detection, and if the concentration was below the lowest detection limit, 50% of the lowest detection value was used (28, 29).

### Definition

Skin change is defined as at least one of the signs: skin color changes, skin eruption, or the development of nodules. Neurological abnormalities encompass changes in consciousness and signs such as involuntary movements, nerve reflexes, and muscle tension.

### Statistical analyses

Normal distribution data were presented as mean ± standard deviation and compared between groups using the independent-samples t-test. Abnormally distributed data were presented as median with interquartile range (IQR) and compared between groups using the Mann-Whitney U test. Categorical variables were expressed as percentage and analyzed using the χ2 test. The RF algorithm was employed to screen for important variables, and the predictive accuracy of prognostics was assessed using a receiver operating characteristic (ROC) curve analysis. Correlations between variables were evaluated using the Spearman correlation test. Linear mixed models were utilized for continuous outcomes and estimated marginal means (EMMs) with a 95% confidence interval (CI) were calculated. Statistical analyses were conducted using SPSS software (version 26.0), and figures were generated using the R programming language (version 4.3.1). All tests were two-sided, and *P* < 0.05 was considered statistically significant.

## Results

### Baseline demographic, laboratory, and clinical characteristics

We enrolled 78 patients with confirmed SFTS in our study. Based on clinical outcomes, they were categorized into a survival group (n=60) and a non-survival group (n=18), resulting in a fatality rate of 23.1% (18/78). **Table 1** presents the baseline demographics, clinical characteristics, and laboratory parameters at admission for both survival and non-survival groups. All SFTS patients had an average age of 65.89 ± 11.19 years. Among these patients, the survivors had an average age of 64.92 ± 11.76 years, while the non-survivors had higher average age of 69.11 ± 8.54 years. Out of the total 78 patients, 37 (47.4%) were male, including 27 (45.0%) in the survival group and 10 (55.6%) in the non-survival group. There was no significant difference in age or gender between the two groups (*P* > 0.05). In comparison to the survival group (925.5, 404.25-2773.25), the non-survival group (3858, 1746.5-4921) exhibited a higher viral load, demonstrating significant differences (*P* < 0.05). In terms of laboratory examination, we observed that alanine aminotransaminase (ALT), aspartate aminotransferase (AST), direct bilirubin (DBIL), blood urea nitrogen (BUN), creatinine (CREA), creatine kinase (CK), creatine kinase isoenzyme MB (CK-MB), α-hydroxybutyrate dehydrogenase (α-HBDH), lactate dehydrogenase (LDH), K^+^, Na^+^, procalcitonin (PCT), activated partial thromboplastin time (APTT), thrombin time (TT), and D-dimer levels were significantly higher in the non-survival group compared to the survival group (*P* < 0.05).All patients showed a decrease in white blood cell (WBC) counts, lymphocytes (LYM), and platelet (PLT) counts, while there was no significant difference between the two groups (*P* > 0.05).

**Table 1:** Baseline demographics, clinical characteristics, and laboratory parameters of patients with SFTS.

Variables	All patients (N=78)	Survivors (N=60)	Non-Survivors (N=18)	*p*-value
Demographics				
Age(years)	65.89 ± 11.19	64.92 ± 11.76	69.11 ± 8.54	0.165
Male	37(47.4%)	27(45.0%)	10(55.6%)	0.432
Total disease duration (d)	13.00(11.00-17.25)	14.00(12.25-18.75)	10.50(8.75-13.25)	**0.003**
Time from onset to admission (d)	5.00(4.00-6.00)	5.00(4.00-6.00)	5.00(3.75-6.00)	0.991
Time of hospitalization(d)	9.00(6.75-13.00)	9.00(7.25-13.00)	6.00(3.75-8.25)	0.058
Highest temperature (°C)	38.71 ± 0.67	38.73 ± 0.71	38.63 ± 0.53	0.570
Viral load (TCID_50_/ml)	1899.00(420.25-3780.50)	925.50(404.25-2773.25)	3858.00(1746.50-4921.00)	**0.005**
Bite by ticks	27(34.6%)	19(31.7%)	8(44.4%)	0.318
Symptoms				
Weak	56(71.8%)	43(71.7%)	13(72.2%)	0.963
Shiver	26(33.3%)	21(35.0%)	5(27.8%)	0.569
Inappetence	30(38.5%)	22(36.7%)	8(44.4%)	0.552
Nausea	26(33.3%)	18(30.0%)	8(44.4%)	0.254
Vomiting	17(21.8%)	10(16.7%)	7(38.9%)	0.093
Diarrhea	18(23.1%)	16(26.7%)	2(11.1%)	0.291
Abdominal pain	10(12.8%)	7(11.7%)	3(16.7%)	0.877
Muscular soreness	22(28.2%)	17(28.3%)	5(27.8%)	0.963
Signs				
Skin changes	5(6.4%)	2(3.3%)	3(16.7%)	0.140
Lymphadenectasis	19(24.4%)	17(28.3%)	2(11.1%)	0.238
Neurological system	12(15.4%)	8(13.3%)	4(22.2%)	0.586
Laboratory examination				
WBC (3.5-9.5*10^9^/L)	2.90(1.76-4.18)	2.81(1.62-4.19)	3.06(2.25-4.41)	0.334
Neutrophils (1.8-6.3*10^9^/L)	1.72(0.93-2.98)	1.63(0.87-2.88)	2.19(1.57-3.24)	0.148
Lymphocytes (1.1-3.2*10^9^/L)	0.64(0.42-0.98)	0.64(0.43-1.00)	0.67(0.38-0.90)	0.695
Monocytes (0.1-0.6*10^9^/L)	0.19(0.07-0.29)	0.18(0.07-0.26)	0.26(0.08-0.44)	0.196
HGB (130-175g/L)	140.05 ± 18.24	138.30 ± 17.44	145.89 ± 20.12	0.122
PLT (125-350*10^9^/L)	59.00(46.75-78.25)	61.00(49.00-82.00)	51.00(42.00-64.75)	0.066
ALT (9-50U/L)	65.00(38.78-134.63)	52.70(38.30-111.78)	133.25(64.90-248.35)	**0.005**
AST (15-40U/L)	151.50(74.20-331.28)	120.25(61.55-205.28)	379.50(170.00-722.53)	**<0.001**
GGT (11-49U/L)	27.00(17.75-66.25)	25.50(17.00-63.50)	39.00(23.96-73.75)	0.245
ALP (40-150U/L)	59.65(51.38-90.78)	58.00(50.38-86.33)	65.30(54.08-108.98)	0.217
Albumin (35-53g/L)	30.92 ± 4.90	31.24 ± 4.85	29.86 ± 5.06	0.295
TBil (2.0-20.4μmol/L)	10.27(7.60-13.04)	9.38(7.50-12.49)	10.67(8.78-19.70)	0.081
DBil (0.1-3.4μmol/L)	3.39(2.36-5.14)	3.22(2.32-4.92)	4.61(2.94-8.26)	**0.030**
BUN (1.7-8.3mmol/L)	6.28(4.19-8.15)	5.34(3.87-6.86)	9.60(7.35-13.70)	**<0.001**
CREA (40-106μmol/L)	66.00(53.23-86.05)	62.60(51.65-71.50)	103.50(69.28-172.38)	**<0.001**
CK (0-190U/L)	497.00(213.25-1347.75)	366.00(183.25-961.75)	1345.00(475.25-2727.50)	**0.006**
CK-MB (0-5ng/mL)	4.02(2.32-10.50)	3.55(1.76-9.73)	7.50(3.50-22.47)	**0.012**
α-HBDH (72-182U/L)	481.51(263.52-584.00)	322.96(241.84-489.29)	609.02(507.60-936.73)	**<0.001**
LDH (80-285U/L)	611.00(370.00-980.25)	522.00(353.25-819.00)	1042.50(714.73-1829.75)	**<0.001**
K^+^ (3.5-5.1mmol/L)	3.68(3.38-4.00)	3.61(3.31-3.87)	4.02(3.49-4.62)	**0.019**
Na^+^(136-146mmol/L)	134.64 ± 4.69	133.97 ± 4.55	136.90 ± 4.55	**0.019**
Hs-CRP (0-5mg/L)	4.07(1.48-12.51)	3.48(1.27-9.46)	10.86(3.12-14.65)	0.081
PCT (0-0.05ng/ml)	0.16(0.10-0.46)	0.14(0.09-0.25)	0.51(0.30-1.04)	**<0.001**
PT (11-14.5s)	12.60(12.00-13.20)	12.60(11.90-13.20)	12.60(12.28-13.30)	0.316
APTT (28-43.5s)	49.85(43.90-58.65)	47.15(43.38-55.60)	58.70(51.23-66.00)	**0.002**
TT (14-21s)	23.85(21.48-27.90)	23.40(21.03-26.20)	29.15(23.38-42.18)	**0.006**
D-dimer (0-0.5μg/ml)	2.67(1.63-4.57)	2.45(1.38-3.42)	5.70(2.49-8.52)	**0.001**

The Categorical variables were presented by frequencies and percentages (n, %), and tested with the χ 2 test. Continuous variables were summarized as means and standard deviations (SD) or as medians and interquartile range (IQR) and tested with t-test or Mann–Whitney U test. P values comparing the group of survival and non-survival.

WBC, White blood cell; HGB, Hemoglobin; PLT, Platelet; ALT, Alanine aminotransferase; AST, Aspartate aminotransferase; GGT, γ-glutamyl transferase; ALP, Alkaline phosphatase; TBiL, Total bilirubin; DBil, Direct bilirubin; BUN, Blood urea nitrogen; CREA, Creatinine; CK, Creatine kinase; CK-MB, Creatine kinase isoenzyme MB; α-HBDH, α-hydroxybutyrate dehydrogenase; LDH, Lactate dehydrogenase; Hs-CRP, High-sensitivity C-reactive protein; PCT, Procalcitonin; PT, Prothrombin time; APTT, Activated partial thromboplastin time; TT, Thrombin time.

Bold values indicate p-values <0.05, statistically different.

### Comparing cytokine levels during the acute phase between the two groups

**Figure 2:**
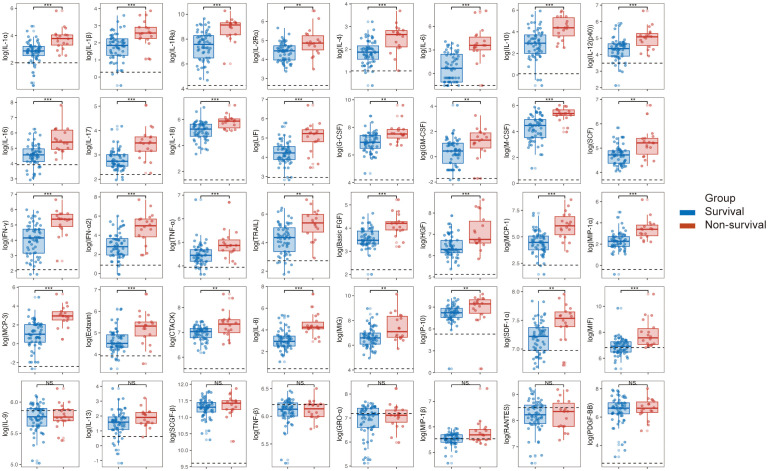
Comparison of cytokine levels between survival and non-survival groups in patients with SFTS in the acute phase. Following the logarithmic transformation of cytokine results, compare the acute-phase cytokine levels between the survival and non-survival groups in SFTS patients. *P < 0.05, **P < 0.01, ***P < 0.001. NS, not significant. IL, interleukin; IFN, interferon; TNF, tumor necrosis factor alpha; LIF, leukemia inhibitory factor; G-CSF, granulocyte colony stimulating factor; GM-CSF, granulocyte macrophage colony stimulating factor; M-CSF, macrophage colony stimulating factor; SCF, stem cell factor; SCGF-beta, stem cell growth factor beta; TRAIL, TNF related apoptosis-inducing ligand; Basic FGF, basic fibroblast growth factor; beta-NGF, nerve growth factor beta; HGF, hepatocyte growth factor; PDGF-BB, platelet derived growth factor-BB; VEGF, vascular endothelial growth factor; CTACK, cutaneous T cell attracting chemokine; Eotaxin, recombinant human eotaxin; GRO-alpha, growth-related oncogene alpha; IP-10, interferon inducible protein 10; MCP, monocyte chemoattractant protein; MIF, macrophage migration inhibitory factor; MIG, monokine induced by gamma interferon; MIP, macrophage inflammatory protein; RANTES, regulated upon activation normal T Cell expressed and presumably secreted; SDF-1alpha, stromal cell derived factor 1alpha.

Due to more than 50% of values being lower limits of detection, 8 cytokines, including IL-2, IL-3, IL-5, IL-7, IL-12 (p70), IL-15, VEGF, and β-NGF, were excluded from the analysis. The results revealed that in the acute phase of SFTS, non-survival group had significantly higher levels of 32 cytokines, such as IL-1α, IL-1β, IL-1ra, IL-2Rα, IL-4, IL-6, IL-10, IL-12(p40), IL-16, IL-17, L-18, LIF, G-CSF, M-CSF, SCF, IFN-γ, IFN-α2, TNF-α, TRAIL, basic FGF, HGF, MCP-1, MIP-1α, MCP-3, Eotaxin, CTACK, IL-8, MIG, IP-10, SDF-1α, and MIF compared to survival group, and these differences were statistically significant (*P* < 0.05). Simultaneously, these cytokine levels were elevated compared to healthy controls, except for MIF in survivors (**Figure 2**).

### MCP-3 serves as a crucial biomarker in predicting the prognosis of SFTS patients

**Figure 3:**
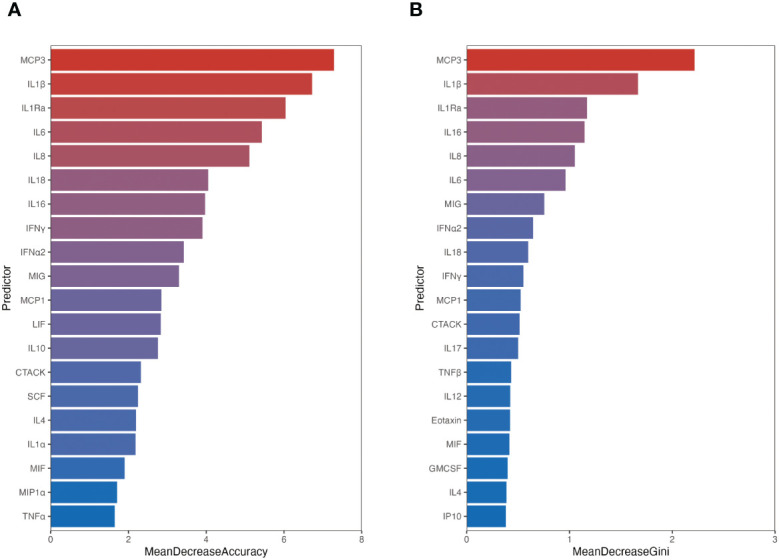
The importance of variables ranked by random forest. Variable importance was ranked according to calculating MeanDecreaseAccuracy **(A)** and MeanDecreaseGini **(B)**. The chart displays the top 20 important variables, with the color bar length indicating the contribution from each variable.

The 78 patients were randomly divided into a training set and a test set at a 7:3 ratio using random seeds with the RF algorithm model. **Supplementary Figure 1A** illustrates the relationship between error and the number of decision trees, with 500 trees chosen as the number of RF models, indicating a stable error. Subsequently, we employed ROC curves to evaluate the predictive ability of the model. The area under curve (AUC) of the ROC for the training set and test set was 1.000 (95% CI: 0.935-1.000) and 0.870 (95% CI: 0.664-0.972), respectively (**Supplementary Figure 1B**). We assessed the importance of 40 cytokines by analyzing the output results of MeanDecreaseAccuracy (**Figure 3A**) and MeanDecreaseGini (**Figure 3B**). The top 20 important variables were then presented, with MCP-3 identified as the most crucial predictive cytokine for the critical outcome of SFTS patients in acute phase.

**Figure 4:**
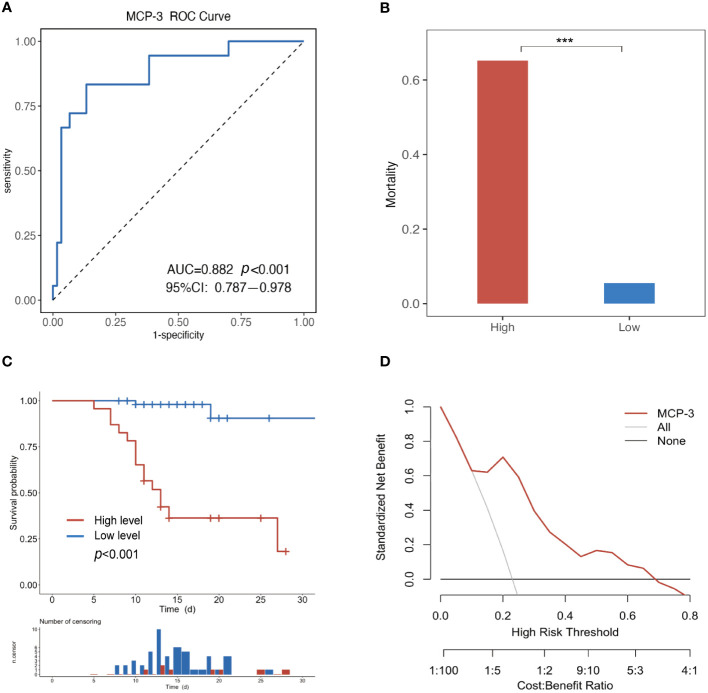
Evaluating the ability of MCP-3 to predict poor prognosis in SFTS patients. **(A)** The MCP-3 had an AUC of 0.882 (*P* < 0.001, 95%CI 0.787-0.978); **(B)** Comparing fatality across different MCP-3 levels. ***P<0.001. **(C)** Kaplan–Meier survival estimate based on the MCP-3 level cut-off; **(D)** Decision curve analysis for the MCP-3. The y-axis represents net benefit, and the x-axis represents the threshold probability (High Risk Threshold). MCP-3, monocyte chemoattractant protein-3; AUC, area under the receiver operating characteristic curve.

Subsequently, ROC curve analysis was employed to assess the specificity and sensitivity of MCP-3 in predicting SFTS patients’ fatal clinical outcomes. MCP-3 had an AUC of 0.882 (95% CI: 0.787-0.978, *P* < 0.001), with a cutoff value of 10.89 pg/ml (**Figure 4A**). Based on the cutoff value, all patients were categorized into low-risk groups, characterized by MCP-3 levels less than or equal to the cutoff value, and high-risk groups, characterized by MCP-3 levels higher than the cutoff value. The fatality rate of the high-risk group (65.2%) was significantly higher than that of the low-risk group (5.5%) (*P* < 0.001) (**Figure 4B**). We further employed the Kaplan-Meier (KM) analysis method to estimate cumulative survival. The results revealed that SFTS patients in the high-risk group, also known as MCP-3 high group, exhibited lower cumulative survival than those in the low-risk group (*P* < 0.001) (**Figure 4C**). Furthermore, the clinical utility of MCP-3 in predicting adverse outcomes in patients with SFTS in the acute phase was evaluated through a decision curve analysis (DCA). As shown in **Figure 4D**, at threshold probabilities between 0.1 and 0.7, the clinical benefit of MCP-3 was markedly superior to that of complete intervention or no intervention at all.

### Correlation analysis of cytokines with laboratory parameters

**Figure 5:**
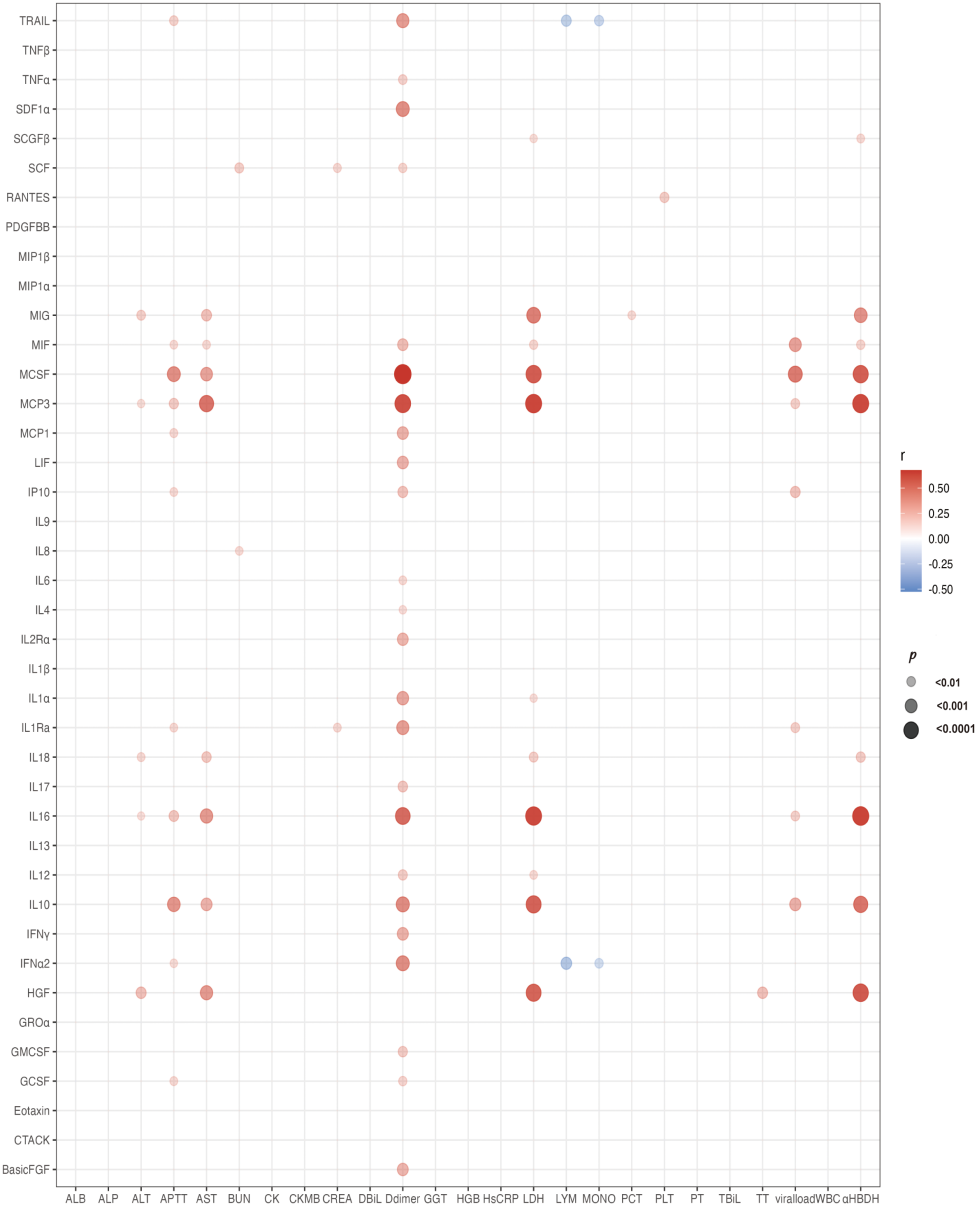
Bubble chart illustrating cytokines associated with laboratory tests. The chart depicts correlations between cytokines and laboratory tests using Spearman correlation analysis. Red bubbles indicate positive correlations, blue bubbles indicate negative correlations. The size of the bubbles represents significant differences, and all bubbles in the graphs indicate significant differences (*P* < 0.01). IL, interleukin; IFN, interferon; TNF, tumor necrosis factor alpha; LIF, leukemia Inhibitory factor; G-CSF, granulocyte colony stimulating factor; GM-CSF, granulocyte macrophage colony stimulating factor; M-CSF, macrophage colony stimulating factor; SCF, stem cell factor; SCGF-beta, stem cell growth factor beta; TRAIL, TNF related apoptosis-inducing ligand; Basic FGF, basic fibroblast growth factor; beta-NGF, nerve growth factor beta; HGF, hepatocyte growth factor; PDGF-BB, platelet derived growth factor-BB; VEGF, vascular endothelial growth factor; CTACK, cutaneous T cell attracting chemokine; Eotaxin, recombinant human eotaxin; GRO-alpha, growth-related oncogene alpha; IP-10, interferon inducible protein 10; MCP, monocyte chemoattractant protein; MIF, macrophage migration inhibitory factor; MIG, monokine induced by gamma interferon; MIP, macrophage inflammatory protein; RANTES, regulated upon activation normal T Cell expressed and presumably secreted; SDF-1alpha, stromal cell derived factor 1alpha; ALT, alanine aminotransaminase; AST, Aspartate aminotransferase; ALP, alkaline phosphatase; BUN, blood urea nitrogen; CREA, creatinine; CK, creatine phosphokinase; CK-MB, creatine kinase isoenzyme-MB; HGB, hemoglobin; PCT, procalcitonin; GGT, γ-glutamyl transferase; LDH, lactate dehydrogenase; PLT, platelet; PCT, procalcitonin; TBIL, total bilirubin; WBC, white blood cell; MONO, monocyte; α-HBDH, α-hydroxybutyrate dehydrogenase; TBIL, total bilirubin; APTT, activated partial thromboplastin time; ALB, albumin; TT, thrombin time; DBIL, direct bilirubin; HsCRP, hypersensitive C-reactive protein.

The correlation analysis of cytokines with laboratory parameters is presented in **Figure 5**. As depicted in the figure, MCP-3 exhibited positive correlations not only with various organ damage parameters, such as ALT, AST, LDH, α-HBDH, APTT, and D-dimer, but also with viral load. From **Figure 5**, it is evident that D-dimer exhibited significant positive correlations with various cytokines, particularly M-CSF, MCP-3, and IL-16. Moreover, LDH and α-HBDH, which are two markers of organ damage, showed positive correlations with various cytokines, especially with M-CSF, MCP-3, MIG, IL-16, IL-10, and HGF.

### Longitudinal cytokine profiles of SFTS patients with different clinical outcomes

**Figure 6:**
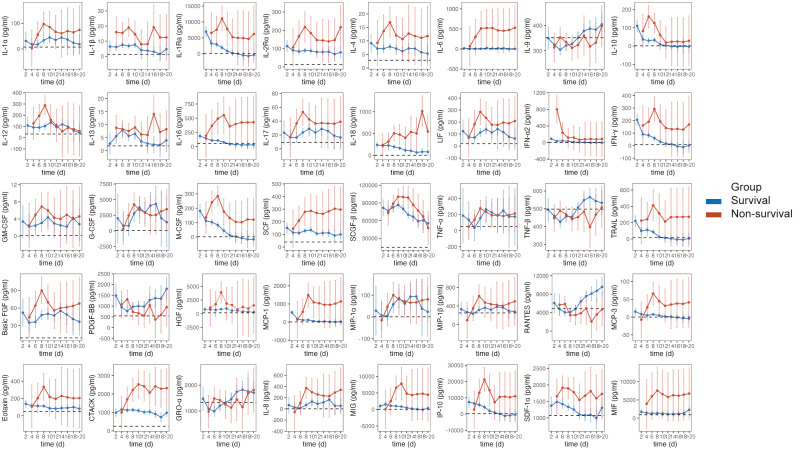
Dynamic evolution of serum cytokines in SFTS patients. Estimated marginal means (EMMs) and 95%CI with linear mixed-effects models for dynamic analysis of cytokines. Error bars indicate 95%CI. The dotted horizontal line within each cytokine panel represents the average value for healthy controls.

In this study, MCP-3 levels exhibited a rapid increase, reaching a maximum, followed by a decrease and stabilization at higher levels until death in non-survivors. These levels consistently surpassed those in survivors and healthy controls. A similar trend was observed for IL-1α, IL-1Ra, IL-4, IL-10, IL-17, LIF, IFN-γ, GM-CSF, M-CSF, SCGF-β, basic FGF, HGF, MCP-1, Eotaxin, MIG, and IP-10. The levels of IL-6, IL-16, IL-18, IFN-α2, HGF, MCP-1, MCP-3, Eotaxin, CTACK, MIG, and MIF were significantly elevated in non-survivors, whereas they remained relatively stable in survivors, comparable to healthy controls. Cytokines such as IL-6, IL-16, IFN-α2, HGF, MCP-1, MCP-3, Eotaxin, MIG, and MIF exhibited similar levels when comparing HCs and survivors. Additionally, we noted lower levels of IL-9, TNF-β, PDGF-BB, and RANTES in non-survivors compared to survivors during hospitalization. Unlike other cytokines, the concentrations of them tended to increase during the late course of the disease in survivors (**Figure 6**).

## Discussion

SFTS is a newly emerging infectious disease with a high mortality rate. The World Health Organization has recognized SFTSV as one of the top-prioritized pathogens for research and development (30). Compared to healthy controls, the study observed that all SFTS patients showed increased levels of various cytokines, except for individual ones such as IL-9, TNF-β, GRO-α, and RANTES. Further analysis indicated that the non-survival group exhibited significantly higher levels of 32 cytokines than the survival group. This finding aligns with previous research, supporting the notion that the immune dysfunction-cytokine storm closely linked to the unfavorable prognosis of SFTS patients (31, 32).

According to the RF algorithm model, MCP-3 has identified as a crucial factor in predicting adverse clinical outcomes in SFTS patients. In non-survivors of SFTS, MCP-3 levels rapidly increased during the acute phase, reaching the highest level. As the disease progressed, MCP-3 concentrations decreased slightly but remained higher than in non-survivors until death. Moreover, compared to healthy controls, the MCP-3 concentration in the patients in the survival group showed a slight increase, but no significant difference observed.

MCP-3 is a member of the monocyte chemotactic proteins (MCP) subgroup of CC chemokines. It was produced by various cells, including monocytes, T lymphocytes, fibroblasts, and platelets (19). MCP-3 is a highly versatile chemokine, activating a broad spectrum of leukocytes, such as natural killer cells and T lymphocytes (33). It is a chemoattractant for monocytes, eosinophils, macrophages, basophils, lymphocytes, and dendritic cells (20). studies have demonstrated the pivotal role of MCP-3 in distinguishing between infectious and non-infectious diseases. Research by Sun L’s team indicates the potential utility of serum MCP-3 levels in distinguishing between patients with mild and severe asthma (21). Yang et al. examined 48 cytokines in the peripheral blood of COVID-19 patients, identifying IP-10, MCP-3, and IL-1ra as biomarkers associated with disease severity and fatal outcomes (34).

Although there is currently no relevant study on the mechanism by which MCP-3 mediates the severity of SFTS, we attempted to analyze it from two perspectives: viral infiltration and inflammatory damage. Our study found that the serum SFTSV level was significantly higher in the non-survival group than in the survival group, and the serum MCP-3 level positively correlated with the SFTSV. CCR2 is recognized as one of the primary receptors of MCP-3 (35). Recent studies have also identified CCR2 as the host receptor of SFTSV. Knocking out CCR2 has been shown to significantly reduce virus binding and infection, while increased expression of CCR2 leads to enhanced infection (23). Hence, it is plausible that MCP-3 assists CCR2 in mediating virus invasion into the host. Additionally, studies have demonstrated that in COVID-19 patients, the increase in viral load corresponds to the upregulation of critical chemokines, including CCL7, CCL2, CXCL8, and others (36, 37).

In terms of inflammation, MCP-3, as an inflammatory cytokine, plays a crucial role in maintaining a balanced level of circulating inflammatory monocytes. A deficiency in MCP-1 or MCP-3 results in approximately 40–50% decrease in monocyte recruitment during infection (38). A study by Girkin J’s team demonstrated that CCL7 is the most significantly upregulated gene induced by rhinovirus infection, and inhibiting CCL7 reduces inflammation and airway hyperreactivity (AHR) (39). Additionally, children with naturally occurring viral infections release high concentrations of MCP-3 and MCP-4 into their nasal secretions (40). To examine the relationship between MCP-3 and organ damage, correlation analysis was conducted on laboratory indicators such as ALT, AST, LDH, α-HBDH, APTT, and D-dimer etc. Previous studies identified these indicators as independent risk factors for poor prognosis in SFTS patients. The findings revealed a significant positive correlation between MCP-3 and these indicators.

In this study, we found that the concentrations of TNF-β had increased in the late stage for survivors, and the related studies were rarely reported in SFTS. There was a study found that TNF-β was considerably increased in patients with COVID-19 (41). Previous studies have shown that the concentrations of RANTES in plasma tended to increase during the late course of the disease, which was consistent with our findings in the survival group (42, 43), similar finding was reported in the late stage of survivors with Middle East respiratory syndrome (MERS) that RANTES marked elevation (44). Our study also suggested that the PLT levels are closely correlated with the levels of RANTES. Another study manifested that RANTES had large fluctuations after onset, and the higher virus loads suppressed the production of RANTES to a large extent (15). RANTES can recruit and activate T cells and they may play a crucial role in recovery from SFTS.

This study has certain limitations. Firstly, the sample size is limited, and the source is singular. Secondly, although we observed the role of MCP-3 in the prognosis of SFTS patients, further experiments are needed to explore its mechanism of action. Lastly, in future research, we aim to expand the sample size and sources, employing sequencing technology to investigate the mechanisms underlying the elevated MCP-3 levels and their impact on patients with severe SFTS.

## Conclusions

In summary, we observed a correlation between abnormally elevated MCP-3 levels and a poor prognosis in SFTS patients. This study is the first to reveal that MCP-3 levels could serve as a meaningful biomarker for predicting the severe and fatal outcome of SFTS patients. These findings contribute to the advancement of our understanding, exploration, and management of SFTS.

## Data availability statement

The raw data supporting the conclusions of this article will be made available by the authors, without undue reservation.

## Ethics statement

The studies involving humans were approved by the Ethics Committee of Beijing Ditan Hospital, Capital Medical University (NO. DTEC-KY2022-022-01) and conducted in accordance with the principles of the Helsinki Declaration. All participants provided written informed consent. The studies were conducted in accordance with the local legislation and institutional requirements. The participants provided their written informed consent to participate in this study.

## Author contributions

ZL: Conceptualization, Formal Analysis, Methodology, Writing – original draft, Writing – review & editing. CZ: Conceptualization, Formal Analysis, Methodology, Visualization, Writing – original draft, Writing – review & editing. HY: Data curation, Methodology, Writing – original draft. RZ: Data curation, Formal Analysis, Writing – original draft. XX: Data curation, Formal Analysis, Writing – original draft. ZJ: Investigation, Resources, Writing – original draft. ZG: Data curation, Writing – original draft. YX: Investigation, Writing – original draft. WZ: Investigation, Writing – review & editing. LL: Supervision, Writing – review & editing. ZC: Conceptualization, Writing – review & editing.
