# Supplementary figures and images for: MCP-3 as a prognostic biomarker for severe fever with thrombocytopenia syndrome: a longitudinal cytokine profile study
Source: Front Immunol. 2024 May 15;15:1379114. doi: 10.3389/fimmu.2024.1379114 (PMC11134196; doi:10.3389/fimmu.2024.1379114)

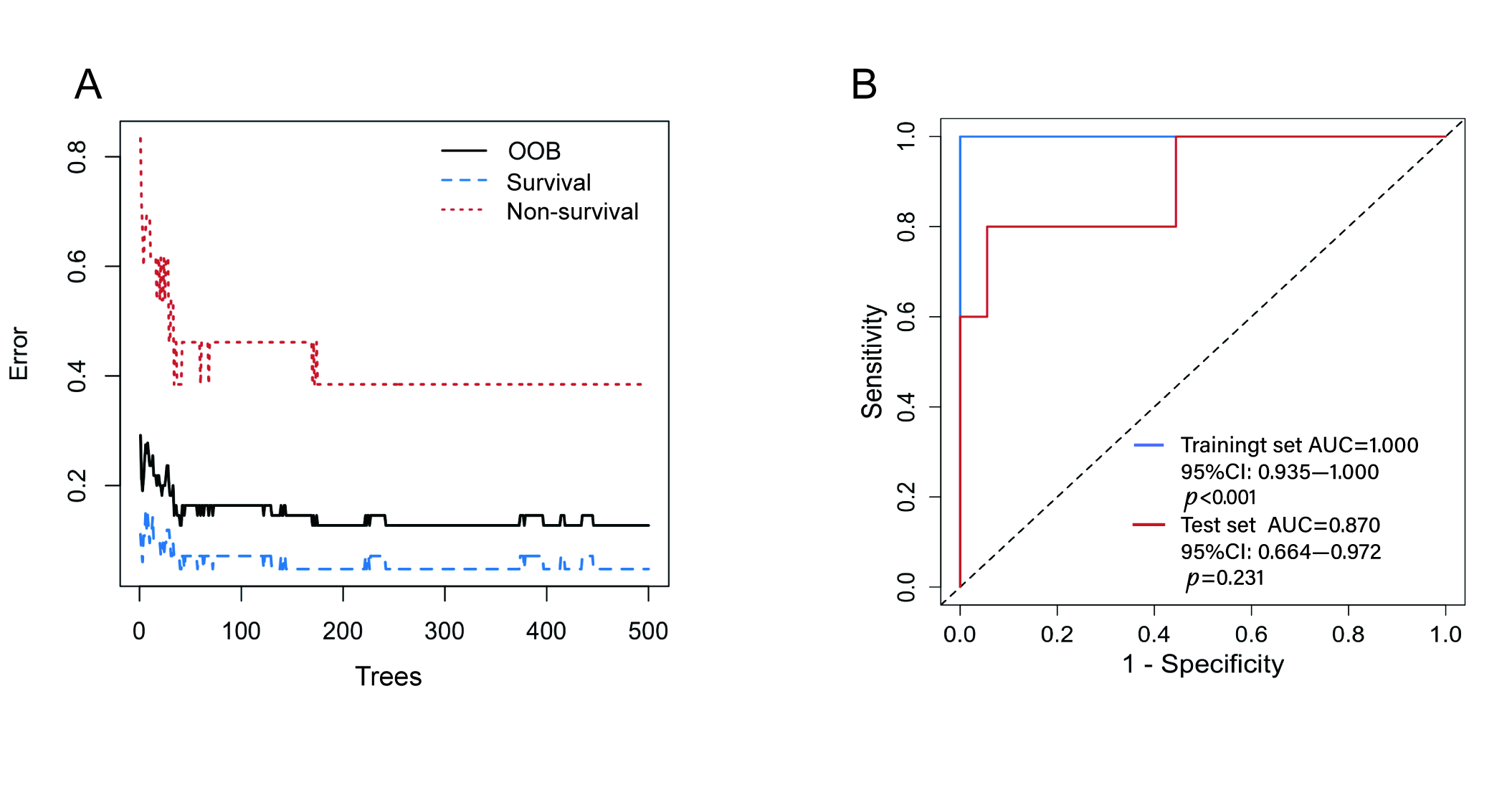

Supplement: Supplementary file 1 [file Image_1.tif]
