# Supplementary material for: MCP-3 as a prognostic biomarker for severe fever with thrombocytopenia syndrome: a longitudinal cytokine profile study
Source: Front Immunol. 2024 May 15;15:1379114. doi: 10.3389/fimmu.2024.1379114 (PMC11134196; doi:10.3389/fimmu.2024.1379114)
Supplement: Supplementary file 2 [file Table_1.docx]

**S1 table.** Comparing the age and sex of SFTS patients with healthy controls.

| **Variables** | All patients  (N=78) | Healthy controls  (N=6) | *p*-  value |
| --- | --- | --- | --- |
| Age(years) | 65.89±11.19 | 64.5±5.43 | 0.766 |
| Male | 37(47.4%) | 3(50%) | 1.000 |

*P* values comparing the group of all patients of SFTS and the healthy controls.
